# Enhancement of femtosecond lenticule extraction for visual symptomatic eye after myopia correction

**DOI:** 10.1186/1471-2415-14-68

**Published:** 2014-05-18

**Authors:** Jing Zhao, Peijun Yao, Zhi Chen, Meiyan Li, Yang Shen, Huamao Miao, Xingtao Zhou

**Affiliations:** 1 The Department of Ophthalmology, Myopia Key Laboratory of the Health Ministry, Eye and ENT Hospital of Fudan University, 83 FenYang Road, Shanghai 200031, China

**Keywords:** Femtosecond lenticule extraction, Enhancement, Myopia, Complication

## Abstract

**Background:**

The novel Femtosecond lenticule extraction (FLEx) procedure has been considered safe, predictable, and effective in treating myopia and myopic astigmatism, with few complications. However, an enhancement procedure after FLEx may be required in some cases, but has not been reported in detail.

**Case presentation:**

A 24-year-old woman who had undergone bilateral FLEx with the VisuMax femtosecond laser treatment for myopic astigmatism complained of double vision in her left eye after the operation. The manifest refraction was −0.50/-1.25 × 180°. The corneal topography showed a central-inferior steepened zone. The ocular wavefront measurements displayed a high value of total aberrations as well as coma. She was scheduled for an enhancement procedure and it was performed by relifting the primary FLEx flap in the left eye four months later. Ablation was made with the Mel-80 excimer laser. After retreatment, the corresponding aberrations were diminished and the corneal topography turned flattened. Her symptom resolved completely with good visual outcomes.

**Conclusion:**

This first detailed case report demonstrates the feasibility and efficacy of enhancement after FLEx for visual symptomatic eye after myopia correction. An analysis of more cases would be necessary to determine a more definite profile.

## Background

In the past several years, the introduction of femtosecond laser technology has brought innovation to corneal refractive surgery as it has been used successfully to create laser in situ keratomileusis (LASIK) flaps [1]. A recent breakthrough of femtosecond laser resulted in a new “all-in-one” refractive procedure called refractive lenticule extraction (ReLEx), which involves two modalities - femtosecond lenticule extraction (FLEx) and small incision lenticule extraction (SMILE). ReLEx has been considered safe, predictable, and effective in treating myopia and myopic astigmatism, with few reports of complications [2]. So far, there is no detailed description of the enhancement after ReLEx. Herein, we present what we believe is the first detailed case report of enhancement after FLEx procedure for visual symptomatic eye after myopia correction.

## Case presentation

**Figure 1:**
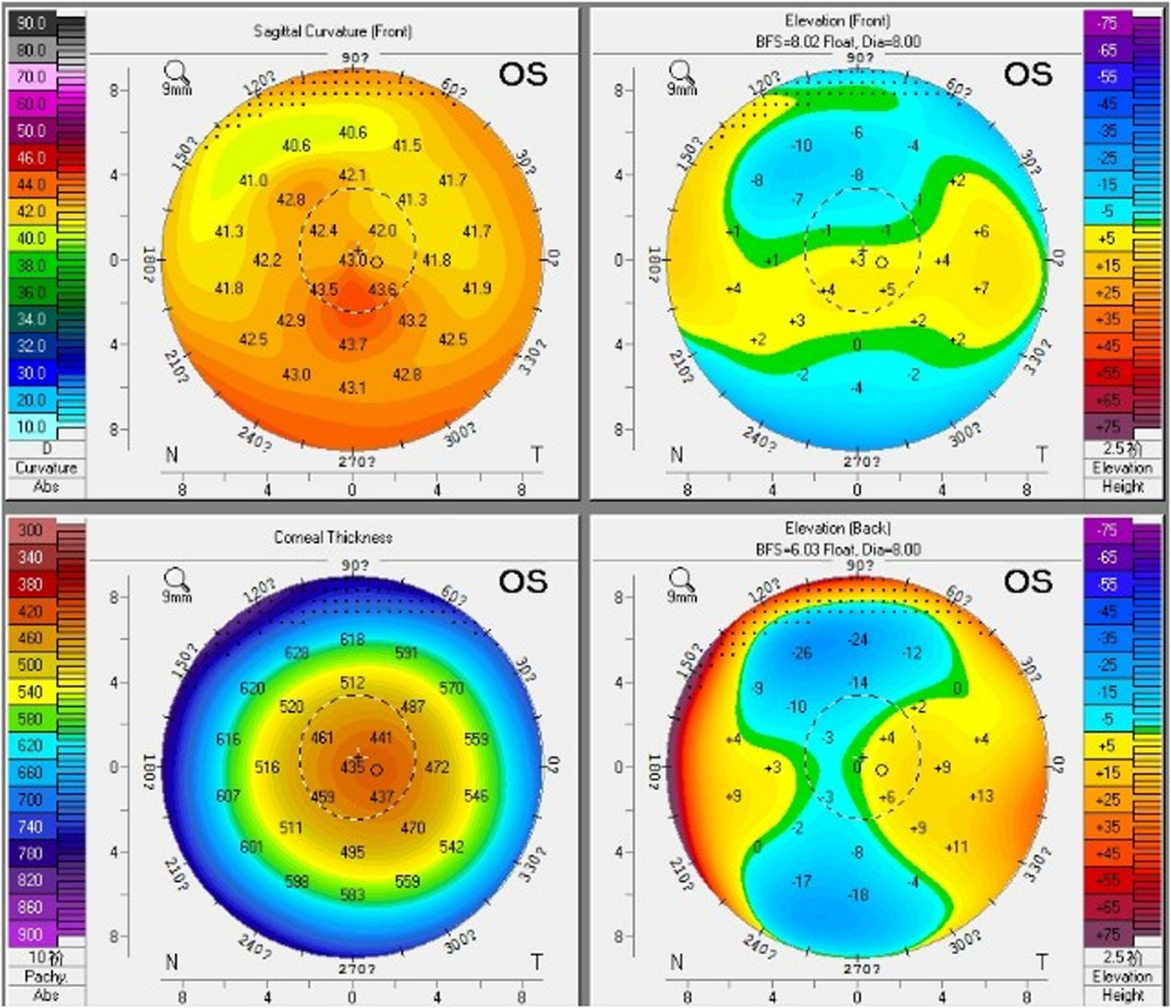
Corneal topography showed a central-inferior steepened zone postoperatively, without significant abnormality of the posterior float.

A 24-year-old woman was referred to our eye clinic complaining of double vision in the left eye after bilateral ReLEx at another refractive surgery center three months prior to consultation. She had undergone uneventful bilateral FLEx, which involves creating and lifting a hinged flap followed by the manual removal of an intrastromal refractive lenticule with the VisuMax femtosecond laser (Carl Zeiss Meditec, Jena, Germany) [2]. The preoperative ophthalmic examinations were normal with the exception of myopia with astigmatism. The manifest refraction was OS −4.25/ -0.75×150° with the corrected distance visual acuity (CDVA) of 20/20. The preoperative corneal central thickness was 534 μm and the mean keratometry reading was 45.7 D. The attempted lenticule thickness was 96 μm, with flap thickness of 100 μm and optical zone of 6.6 mm. In the early postoperative period, the patient complained of constant double vision extending laterally and vertically around the visual target. This disturbance was experienced in the left eye only, and her perception was worst in dim environment. The routine postoperative ophthalmic examinations were performed at our clinic. The uncorrected distance visual acuity (UDVA) was 20/25 in the left eye and the manifest refraction was −0.50/-1.25 × 180° with a corrected distance visual acuity (CDVA) of 20/20. Ocular wavefront aberrations were measured with COAS analyzer (Carl Zeiss, Meditec AG, Germany) and analyzed at 5 mm pupil size using 6 orders of Zernike polynomials. The root mean square (RMS) values of total aberrations, higher order aberrations and coma were 1.44 μm, 0.52 μm and 0.31 μm in the left eye, respectively. The corneal topography was measured by Scheimpflug imaging (Pentacam; Oculus, Wetzlar, Germany), showing a central-inferior steepened zone, without significant abnormality of the posterior float (Figure 1). The left eye was treated with fluorometholone 0.1% drops four times daily for two weeks. One month later, the UDVA was at 20/20 but the double vision symptom was not alleviated. Furthermore, the central-inferior steepened zone remained unchanged and the manifest refraction was stable.

**Figure 2:**
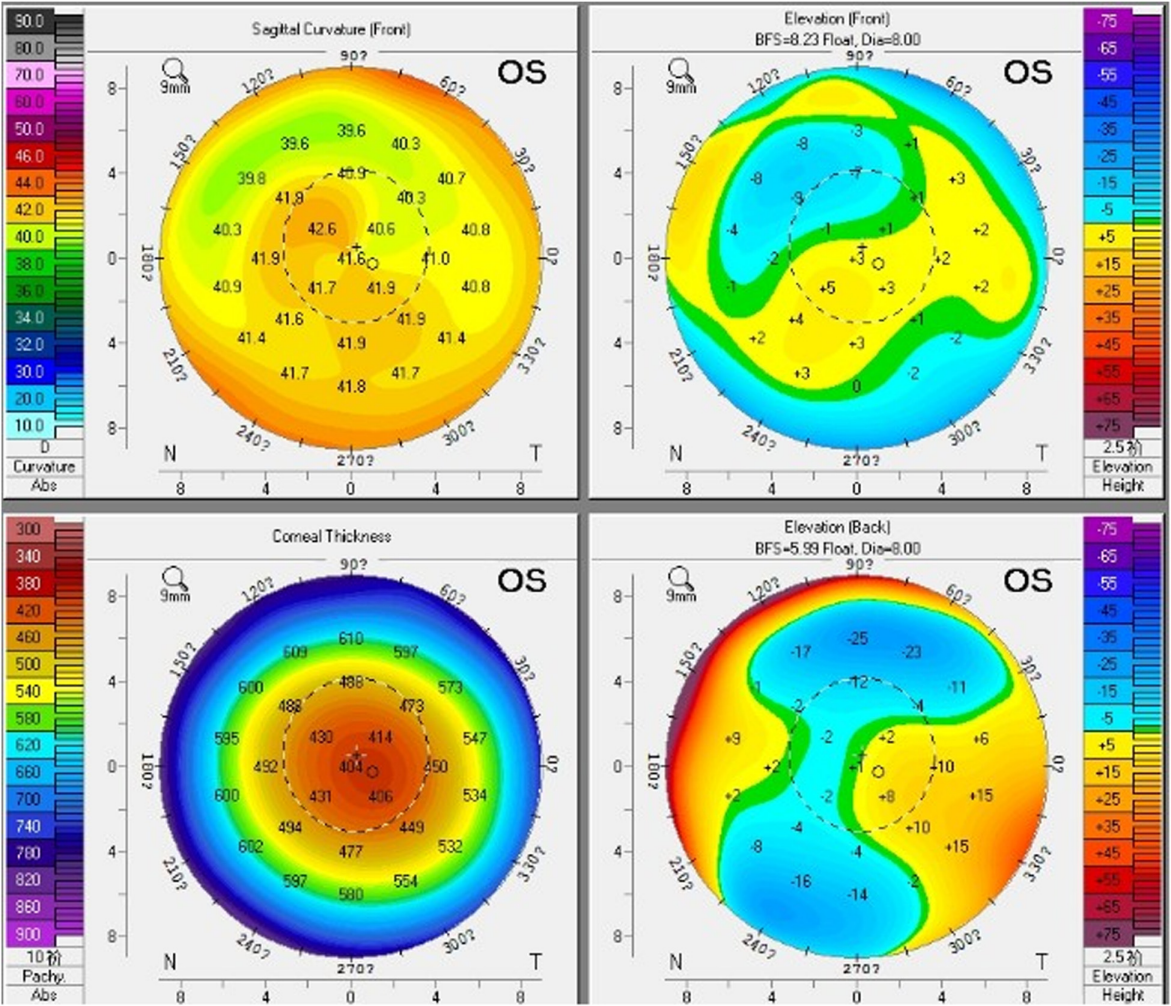
Corneal topography showed a central flattened surface and remained stable in 1 month post-enhancement.

**Figure 3:**
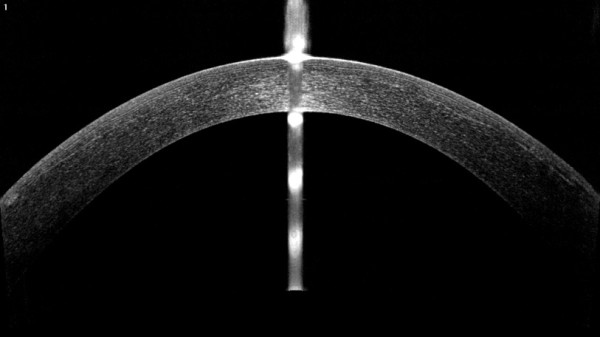
Fourier-domain OCT image showed a uniform flap well attached to the stromal bed and no interface fluid or epithelial ingrowth were noted in the left eye at 5 weeks post-enhancement.

At four months postoperatively, the patient was scheduled for an enhancement procedure in the left eye. A Sinskey hook was used to undermine the edge of the flap inferiorly, separating the flap from underlying stromal bed. After the lift edge has been extended to 5 mm, a non-toothed forceps was used to lift the flap gently from its stromal bed and peeled back superiorly towards the hinge. Then the excimer laser ablation was performed, using the Mel-80 excimer laser, which is a conventional platform (Carl Zeiss Meditec AG, Germany) for a correction of −0.50/-1.25 × 180° with intended stromal ablation of 38 μm and the optical zone was set 6.75 mm. Irrigation of the interface with balanced salt solution was carried out, followed by flap reposition. A silicon-hydrogel bandage contact lens was applied immediately after surgery and removed the following day. Postoperative medication was prescribed for the operated eye. Five weeks after the enhancement, the UDVA was 20/20 and the subjective refraction was plano with a CDVA of 20/20. Under slit-lamp examination, the flap was intact with no flap striae, inflammation, epithelial ingrowth, or diffuse lamellar keratitis (DLK). The RMS values of total aberrations, higher order aberrations, and coma of 0.77 μm, 0.47 μm and 0.18 μm were all diminished after retreatment. The corneal topography showed a central flattened surface and remained stable within one month of the post-enhancement (Figure 2). Fourier-domain OCT showed a uniform flap that was attached well to the stromal bed and no interface fluid or epithelial ingrowth were noted in the left eye (Figure 3). The patient reported that double vision had resolved completely and she was extremely satisfied with the enhancement.

## Discussion

Similar to LASIK, an enhancement procedure after ReLEx may be required in some cases as the refractive outcome might be dependent on various factors such as wound healing, the extent of original refractive status, patient expectations, as well as surgeon treatment philosophies and techniques [3]. To our knowledge, this is the first detailed description of enhancement after FLEx.

In our case, the postoperative corneal topography showed a steepened surface centrally and inferiorly, which remained stable within four months. This may rule out the possibility of corneal edema or interface hydrops. We assumed this steepened zone might attribute to the incomplete separation or extraction of the lenticule during the manual operation, which might be caused by imperfect laser scan, or an unexpected decentration occurring during suction and scanning. A bit of residual intrastromal lenticule tissue which has not been detected might still remain in the corresponding zone, which could cause irregular corneal topography, high RMS value of ocular aberration and astigmatism. The indications for enhancement of LASIK are residual myopia, hyperopia, or astigmatism greater than 0.75 D, with evidence of refractive stability [3]. According to these indications, it seemed necessary to perform an enhancement procedure on this patient to improve her visual outcomes. The patient’s main complaint after FLEx was double vision. It has been demonstrated that aberrations can be the cause of various symptoms postoperatively and different symptoms may correspond with certain aberrations. Chalita *et al.*[4,5] found that double vision correlates with total coma but not spherical aberration under scotopic conditions. Their observations suggested that the relief of double vision in the retreated eye is primarily associated with the reduction of coma as it is in this case.

Enhancement after LASIK can be performed safely and effectively by relifting the primary LASIK flap, creating a new flap (recutting) as well as surface ablation before excimer laser ablation [6]. It has been demonstrated that the lifting of the flap should generally be undertaken within three months after the initial LASIK procedure [6]. In our case, we performed the enhancement four months after the previous FLEx by relifting the flap with ease, which was confirmed by Santhiago *et al*. [7] who implied that relifting of the flap may be performed without difficulty within one year of the femtosecond laser-assisted LASIK surgery. Though lifting the original LASIK flap has been proven to show better long term stability and less chance of flap complications than recutting [8], it may increase the risk of epithelial ingrowth, Diffuse lamellar keratitis (DLK), flap melting, striae and flap torn [6,9]. However, none of these complications were observed in our case. Surface ablation could be taken into account as well for such case as it might correct more of the corneal abnormality, especially when the irregularity is in the flap itself rather than the stromal bed. Albeit successful retreatment after FLEx performed in our case, the enhancement of small incision lenticule extraction (SMILE) [10], which is the other modality of ReLEx should be paid close attention, too. As no flap is made in SMILE procedure, creating a new flap or surface ablation are the available options to be considered. Further investigation of the enhancement would be necessary to evaluate clinical outcomes and visual quality to determine which approach is the best.

## Conclusion

In conclusion, our analysis of the current case demonstrates the feasibility and efficacy of enhancement after previous FLEx for visual symptoms. More cases and further studies will be necessary to determine a more definite profile.

## Consent

Written informed consent was obtained from the patient for publication of this Case report and any accompanying images. A copy of the written consent is available for review by the Editor of this journal.

## Abbreviations

ReLEx: Refractive lenticule extraction; FLEx: Femtosecond lenticule extraction; SMILE: Small incision lenticule extraction; DLK: Diffuse lamellar keratitis.

## Competing interests

The authors declare that we have no competing interests.

## Authors’ contributions

JZ conceived of the study and drafted the manuscript. PJ Y, ZC, XT Z critically revised the manuscript. XT Z performed the surgery, JZ, MY L, YS, HM M, ZC, PJ Y performed the examination, collected the data, and helped to draft the manuscript. All authors participated in the design of the study, read and approved the final manuscript.

## Pre-publication history

The pre-publication history for this paper can be accessed here:

http://www.biomedcentral.com/1471-2415/14/68/prepub
